# Hypoacusia in a Patient Treated by Isotretinoin

**DOI:** 10.1155/2011/789143

**Published:** 2011-11-24

**Authors:** L. Rosende, M. M. Verea-Hernando, A. de Andrés, F. Piñeyro-Molina, J. Barja, S. Castro-Castro, E. Fonseca

**Affiliations:** ^1^Department of Dermatology, Complexo Hospitalario Universitario A Coruña, Hospital Abente y Lago, 15001 A Coruña, Spain; ^2^Department of Otorhinolaryngology, Complexo Hospitalario Universitario A Coruña, 15006 A Coruña, Spain

## Abstract

Isotretinoin is the most effective treatment for severe acne, but there are several adverse effects associated with its use, some of them very exceptional (<1/10000). We report one case of hypoacusia and tinnitus in a 15-year-old boy treated with isotretinoin during 6 weeks, who quickly improved after isotretinoin withdrawal. Also, we comment other publications about hearing alterations in patients treated with isotretinoin and other retinoids.

## 1. Introduction

 Isotretinoin is the most effective treatment for severe acne. Several adverse effects have been reported since its approval by the Food and Drug Administration (FDA) in 1982. Cheilitis and dry skin [1] are the most common, but hearing alterations are exceptional (<1/10000) [2]. We report one case of hypoacusia and tinnitus in a boy treated with isotretinoin, who quickly improved after isotretinoin withdrawal.

## 2. Case Report

 A 15-year-old boy with severe nodulocystic acne on his face and back, who failed to respond to conventional therapy, was treated by oral isotretinoin at a daily dose of 30 mg (0.5 mg/kg). At one month followup, the physical examination only evidenced cheilitis and dry skin, and a blood test was normal. Two weeks later, he started with bilateral tinnitus and hypoacusia. There was no history of previous trauma or other concomitant drug intake and he denied fever, headache, dizziness, nausea, or vomiting. Neurological examination was unremarkable. 

The examination by an otorhinolaryngologist determined a normal eardrum and external ear conduct, a Rinne test positive, and a Weber test without lateralization. The verbal acumetric estimated a hearing loss of 45 db according to Fowler's table, more evident for the words containing acute vowels (i, e, a), which estimates losses from 500 to 3000 Hz. 

Due to a suspected diagnosis of isotretinoin-caused ototoxicity, this drug was immediately discontinued and an audiometry was requested. The patient reported a rapid improvement after isotretinoin withdrawal, and the audiometry made 15 days later only showed minimal alterations.

## 3. Discussion

 Some studies have shown the influence of isotretinoin on the ear. In 1988, in one of the 104 reports of suspected adverse reactions to isotretinoin, Bidgy and Stern found decreased hearing [3]. Nikiforidis et al. [4] found subclinical changes in auditory brainstem response in 9% of the patients treated during 3 weeks with isotretinoin and considered reasonable suggest these subclinical changes may be due to an isotretinoin-induced synaptic malfunction or to a conduction defect in the auditory nerve fibers. Also, a prospective study of 32 patients found significant modifications in brainstem auditory and visual evoked potentials after isotretinoin administration [5]. On the other hand, a recent study of 38 patients with acne vulgaris treated with isotretinoin found decreased hearing thresholds of the patients after one, two, and three weeks of treatment [6]. 

 In regard to other retinoids, one case of deafness with acitretin was published [7]. This patient noticed sudden bilateral sensorineural hearing loss with tinnitus after one week of treatment, and she improved after reducing dose. Otherwise, in a study of 12 cases of acitretin therapy for hidradenitis suppurativa [8], one patient developed tinnitus after 4 months with the treatment. Tinnitus disappeared with the withdrawal, and repeated after reintroduction of the treatment, and also developed headache and lack of concentration, so treatment was discontinued. 

In our case, the relationship between ototoxicity and isotretinoin is probable with a score of 5 points with the Naranjo probability scale [9]. The adverse event appeared after isotretinoin was administered, improved when the drug was discontinued, and there were no other causes of hearing loss. We have not any objective evidence other than the otologic examination, because the audiometry was made when the patient had improved, two weeks after withdrawal. 

 If a patient treated with isotretinoin develops tinnitus or deafness, it seems reasonable think that the drug could induce nerve conduction alteration, so a complete exploration including audiometry and auditory evoked potentials could be indicated, but trying not to delay the withdrawal of the drug.
